# Hypothyroidism and multinodular goiter due to tubercular thyroiditis: A rare coexistence

**DOI:** 10.1016/j.amsu.2022.104724

**Published:** 2022-09-16

**Authors:** Achraf Amine Sbai, Najat Draoui, Adil Abdenbi Tsen, Fahd Elayoubi

**Affiliations:** a Department of Ear Nose and Throat, Mohammed VI University Hospital, Medical School, Mohammed the First University, Oujda, Morocco; b Laboratory of Epidemiology, Clinical Research and Public Health, Faculty of Medicine and Pharmacy of Oujda, Mohammed the First University, Morocco; c Endocrinology and Diabetology Departement, Morocco; d Department of Cervicofacial Surgery, Mohammed VI University Hospital, Medical School, Mohammed the First University, Oujda, Morocco

**Keywords:** Tuberculosis, Thyroid, Giganto-cellular granulomatous

## Abstract

**Introduction and importance:**

the diagnosis of thyroid tuberculosis is often difficult, on account of its rarity even in countries with endemic tuberculosis, and on account of its non-specific clinical, biological and radiological presentation.

**Case presentation:**

A 38-year-old woman presented with swelling in the anterior part of the neck for 7 years. Clinical and radiological examination found multinodular goiter and fine needle aspiration cytology showed colloidal cells with follicular cells. A total thyroidectomy was performed and histological examination of showed epithelioid and giganto-cellular granulomatous with caseous necrosis, confirming the diagnosis of tuberculous thyroiditis.

**Clinical discussion:**

Tuberculosis of the thyroid gland is a very rare disease, the diagnosis is often made by fine needle aspiration cytology (FNAC), the treatment is mainly medical with antituberculosis drugs, but surgery remains a therapeutic means for some cases.

**Conclusion:**

The diagnosis of thyroid TB should be suspected in the presence of a thyroid swelling or nodule, especially in countries with a high prevalence of TB, to allow for early and appropriate management.

## Highlights

• Thyroid tuberculosis is a rare entity even in endemic countries.
• The association of thyroid tuberculosis and a multinodular goiter is even rarer.
• While the association of tuberculous thyroid disease and hypothyroidism is exceptional.
• We report our experience of the management of thyroid tuberculosis which is added to the limited data of the literature.

## 1 Introduction

Tuberculosis in the thyroid gland is a very rare disease, that can be primary or can be part of the generalized tuberculosis. Although TB in the thyroid gland is rare, not exceeding 0.1–0.4%, its incidence is increasing due to the systematic practice of fine needle aspiration cytology (FNAC), which has allowed adequate management.

However, the diagnosis of thyroid TB remains difficult due to its polymorphous characteristics, whether clinical (adenoma, goiter, abscess), biological (euthyroidism, hyperthyroidism or hypothyroidism) or radiological (nodule, goiter).

The management of thyroid TB is currently based essentially on antituberculosis drugs, whereas recourse to surgical treatment is rarer [1,2].

## 2 Case report

A 38-year-old woman who presented with a swelling over the anterior midline of the neck for the last 7 years. The swelling progressively increased in size. Throughout these 7 years the patient presented a weight gain, cold intolerance, and constipation, while as pressure symptoms she does not present hoarseness of the voice or dysphagia. The patient is low socioeconomic status, lives in a small town, with no previous pathological history, suffers from cancerophobia, and the size of the goiter is aesthetically disturbing to her. Although the patient complained of night sweats without fever, chest pain or haemoptysis, it should be noted that the patient did not have any past history of tuberculosis.

On physical examination, she had diffuse, mildly tender thyromegaly. The swelling was soft to firm in consistency, moved with swallowing, with an irregular surface and edges that were not well defined. The overlying skin was indurated. The cervical or axillary lymph nodes were not palpable, other systemic and regional examinations revealed no abnormalities.

Routine hematological and biochemical investigations like blood sugar, urine examination and serum electrolytes of the patient were normal, the thyroid-stimulating hormone was elevated and free T4 were lower, the antithyroid peroxidase antibodies were normal. No tracheal shift was detected in the chest radiograph and electrocardiogram was normal. Nasal Endoscopy showed both the vocal cords to be freely mobile.

Ultrasound (USG) of the thyroid gland revealed an enlarged thyroid gland (3.3 × 5.7 × 7.9 cm) with multiple echogenic nodules of varying size, without features of malignancy. Color Doppler showed increased vascularity of the gland, USG also showed absence of lymph nodes.

Two fine FNAC were performed, the first one was non-diagnostic and the second one was showed colloidal cells with follicular cells without any evidence of malignancy. Based on the clinical and biological findings, USG and FNAC, a diagnosis of multinodular colloid goiter was made. In view of these data the patient underwent a total thyroidectomy after normalization of thyroid hormones by substituting hypothyroidism with Levothyroxine (75 μg per day).

**Fig. 1:**
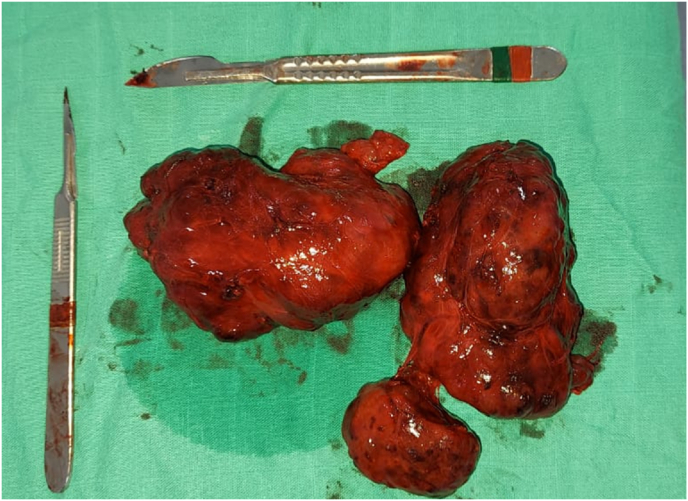
TThe surgical specimen of the total thyroidectomy.

Intraoperatively, the thyroid gland was found to be enlarged with multiple nodules involving both lobes. With absence of adhesions between the surface of the thyroid gland and the surrounding muscles, the recurrent and laryngeal nerve were identified preserved on both sides. Macro and normovesicular multinodular thyroid goiter, reworked with hemorrhagic foci (Fig. 1).

**Fig. 2:**
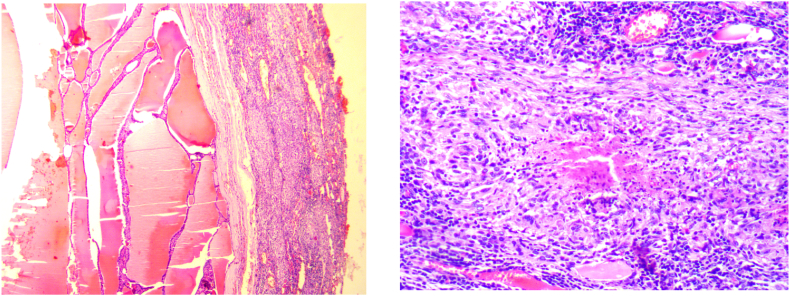
(A,B): The thyroid tissue contains multiple epithelioid and gigantocellular granulomas. These granulomas are of different sizes, confluent, sometimes centered with caseous necrosis. They are also surrounded by a lymphocytic crown.

Epithelioid and giganto-cellular granulomatous thyroiditis with caseous necrosis, no histological evidence of malignancy. Hence, a final diagnosis of tuberculosis of thyroid was made (Fig. 2). The patient has not received anti-tuberculosis treatment because she has no other tuberculous focus and has undergone total thyroidectomy. At the last visit the patient is asymptomatic and euhyroid under substitution by Levothyroxine (100 μg/day). Our study has been reported following the SCARE 2020 Checklist criteria [3].

## 3 Discussion

Tuberculosis of the thyroid gland may be sporadic/primary by direct infection of the gland by the bacillus, or secondary by generalized dissemination from other infected organs, whether pulmonary or extrapulmonary such as a cold abscess.

The presence of tuberculosis in the thyroid gland, whether primary or secondary, remains a rare entity. This can be explained by the importance of the thyroid vascularization, the high concentration of iodine, the phagocytic action and the antibacterial activity of the colloidal material. Due to the rarity of thyroid TB, all available information is the result of isolated case reports. Cases may present with swelling and pressure symptoms such as dysphagia, dysphonia, or hoarseness of the voice due to nerve paralysis, as a result of compression or fibrosis of the surrounding structures. Most commonly, thyroid TB presents as a solitary thyroid nodule. Less commonly, it presents as subacute thyroiditis, chronic thyroiditis, or an acute or cold abscess In our case, the TB presented as a multinodular goiter without any other pulmonary or extrapulmonary involvement [1. 4].

The non-specific symptoms of thyroid TB make her diagnosis difficult to establish. In most cases the thyroid function remains intact, whereas in the literature there are rare cases of thyroid TB with hyperthyroidism or hypothyroidism due to destruction of the thyroid by tuberculosis [1]. Abhideep Chaudhary et al. reports a case of a 40 year old woman with thyroid TB with hypothyroidism on thyroid hormone supplementation 4 years before the diagnosis of thyroid TB.

Our patient presents as a symptom of thyroid TB the isolated night sweats, while the reason that pushed him to consult was the goiter and the symptoms of hypothyroidism [5].

The presence on the cytological examination (the FNAC) of epithelioid granulomas with central necrosis or on the histopathological examination (operating specimen) of epithelioid granulomas or acid-alcoholic bacilli (BAA) in the tissues, allowed the diagnosis of tuberculosis thyroid [4].

However, epithelioid and giant cell granulomas make the diagnosis of thyroid tuberculosis, especially since BAA are not always found in histologic specimens. While the presence of caseous necrosis in granulomas is an important feature that differentiates the diagnosis of thyroid TB and sarcoidosis.

In our patient, cytology did not allow the diagnosis, whereas granulomatous inflammation with acid-alcohol bacilli was observed on histological examination (the surgical specimen), which allowed the diagnosis of tuberculosis. [4.5].

Treatment of tuberculosis of the thyroid gland is based on anti-tuberculosis drugs and/or surgery. Previously the management was based on medical treatment combined with surgical drainage and resection of the affected parts, so nowadays antituberculosis drugs considered as the first line treatment modality that can be sufficient without recourse to surgery [2,6].

For our patient, cytology did not allow the diagnosis, and by evaluating the clinical, biological and radiological data, a thyroidectomy was performed. Histological examination of the surgical specimen showed granulomatous inflammation with acid-alcohol bacilli, which led to the diagnosis of thyroid tuberculosis.

In patients who have undergone total thyroidectomy, antituberculosis treatment for at least 6 months is given if additional foci of tuberculosis are present, otherwise close follow-up without antituberculosis administration is recommended if no additional foci are detected. However, if thyroid tuberculosis was found on histological examination of a surgical specimen from a subtotal or near-total thyroidectomy or lobectomy, 6 months of anti-tuberculosis treatment is recommended even if no additional foci are detected. Our patient did not receive antituberculosis treatment because she has no other tuberculous focus and underwent a total thyroidectomy [4,6].

## 4 Conclusion

It is known that TB of the thyroid gland is a very rare entity, especially the coexistence of TB with a goiter, even the diagnosis remains difficult to establish and requiring a cytological or histological proof, but it is always necessary to think of thyroid TB especially in pandemic countries.

## Provenance and peer review

Not commissioned, externally peer-reviewed.

## Availability of data and material

The datasets in this article are available in the repository of the ENT database, Chu Mohamed VI Oujda, upon request, from the corresponding author.

## Sources of funding

This research was not funded.

## Ethical approval

This is a case report that does not require a formal ethical committee approval. Data were anonymously registered in our database. Access to data was approved by the head of the department.

## Consent

Written informed consent was obtained from the patient for publication of this case report and accompanying images. A copy of the written consent is available for review by the Editor-in-Chief of this journal on request.

## Author contributions

Dr. Achraf Amine SBAI and Najat DRAOUI wrote the manuscript.

Pr. Adil Abdenbi Tsen helped in writing and literature review.

Pr. Fahd ELAYOUBI helped in writing, supervised the redaction, revised and approved the final draft for publication.

All authors approved the final version of the manuscript.

## Registration of research studies

This is not an interventional study. We only reported the patient's findings from our database as a case report.

## Guarantor

Dr Achraf SBAI.

## Declaration of competing interest

The authors declare no conflicts of interest.
